# Severe Hyperferritinemia in Metabolic Dysfunction-Associated Steatotic Liver Disease With Normal Transferrin Saturation and Polyclonal Hypergammaglobulinemia Mimicking Iron Overload

**DOI:** 10.7759/cureus.109279

**Published:** 2026-05-20

**Authors:** Niyas Khalid Ottu Para, Georgey Koshy, Seema Rab

**Affiliations:** 1 Internal Medicine, Burjeel Hospital, Abu Dhabi, ARE; 2 Cardiology, Burjeel Hospital, Abu Dhabi, ARE; 3 Internal Medicine, Burjeel Holdings, Abu Dhabi, ARE

**Keywords:** hyperferritinemia, insulin resistance, metabolic dysfunction-associated steatotic liver disease (masld), polyclonal hypergammaglobulinemia, primary iron overload

## Abstract

Metabolic dysfunction-associated steatotic liver disease (MASLD) is increasingly recognized as a systemic immunometabolic disorder characterized by the interplay between insulin resistance, hepatic lipid accumulation, and chronic low-grade inflammation. While mild elevations in serum ferritin are frequently observed in MASLD, markedly elevated ferritin levels often prompt evaluation for iron overload syndromes, inflammatory disorders, or malignancy. The coexistence of polyclonal hypergammaglobulinemia further complicates diagnostic interpretation.

We report a 36-year-old female with overweight status, insulin resistance (homeostasis model assessment of insulin resistance (HOMA-IR) = 6.3), prediabetes (glycosylated hemoglobin = 6.1%), hypertriglyceridemia, and hyperuricemia, who demonstrated persistent hyperferritinemia (1281-1642 ng/mL) over 19 months with normal transferrin saturation (23%). Liver enzymes were mildly elevated, while bilirubin, gamma-glutamyl transferase, lactate dehydrogenase, C-reactive protein, and renal function remained normal. Serum protein electrophoresis revealed polyclonal hypergammaglobulinemia with elevated IgG. Imaging confirmed severe hepatic steatosis (S3) without fibrosis. MRI-based liver iron quantification demonstrated normal liver iron concentration (LIC = 2.70 mg/g), definitively excluding iron overload.

Extensive evaluation excluded iron overload, autoimmune disease, and hematologic pathology. This case illustrates a clinically important pattern within MASLD in which hyperferritinemia reflects inflammatory signaling and iron sequestration rather than iron overload. Recognition of this phenotype enables confident diagnosis and prevents unnecessary investigations and inappropriate therapy.

## Introduction

Metabolic dysfunction-associated steatotic liver disease (MASLD) represents the hepatic manifestation of systemic metabolic dysfunction and is closely linked to insulin resistance, dyslipidemia, and adipose tissue dysregulation [1,2]. At the cellular level, increased free fatty acid flux to the liver results in triglyceride accumulation, lipotoxicity, mitochondrial dysfunction, and activation of inflammatory signaling pathways, including nuclear factor-kappa B (NF-κB) and Janus kinase/signal transducer and activator of transcription (JAK-STAT) cascades [3].

Ferritin, traditionally viewed as a marker of iron stores, is increasingly recognized as an acute-phase reactant regulated by inflammatory cytokines and oxidative stress pathways [4,5]. Consequently, ferritin elevation may reflect metabolic and inflammatory signaling rather than true iron overload.

Polyclonal hypergammaglobulinemia represents non-specific activation of the adaptive immune system and is typically associated with chronic inflammatory states. Its presence in MASLD reflects a broader immunometabolic activation state involving crosstalk between hepatic innate immunity and systemic immune responses.

While mild to moderate hyperferritinemia is well described in MASLD, the occurrence of persistently elevated ferritin exceeding 1000-1500 ng/mL in conjunction with polyclonal hypergammaglobulinemia introduces significant diagnostic ambiguity. This pattern often leads to evaluation for iron overload syndromes, autoimmune disease, or malignancy. The present case highlights this clinically relevant phenotype and provides a mechanistic framework for its interpretation.

## Case presentation

A 36-year-old female presented with a two-year history of fatigue, dizziness, intermittent nausea, and headaches. There was no history of alcohol consumption. Over the preceding 19 months, she had been noted to have persistently elevated ferritin levels.

Her anthropometric profile showed a body mass index of 26.4 kg/m² (weight = 62 kg, height = 152 cm), with blood pressure of 129/80 mmHg. The patient reported regular menstrual cycles without menorrhagia or abnormal uterine bleeding. She was on escitalopram 20 mg, clonazepam, omega-3 fatty acids, dapagliflozin-metformin combination therapy, and fenofibrate.

Serial laboratory evaluation demonstrated persistent hyperferritinemia ranging from 1281 to 1642 ng/mL. Fasting transferrin saturation remained normal at 23%, with normal serum iron, transferrin, and total iron-binding capacity. Serial ferritin measurements demonstrated persistently marked elevation over a 19-month period without progressive biochemical evidence of systemic iron overload. Serial ferritin trends are illustrated in Figure 1.

**Figure 1:**
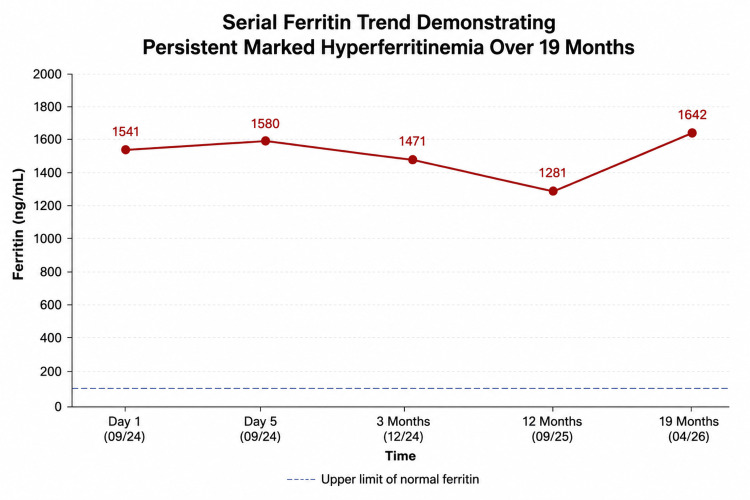
Serial ferritin trend demonstrating persistent marked hyperferritinemia. The figure demonstrates persistently elevated ferritin levels ranging between 1281 and 1642 ng/mL over approximately 19 months. The dashed horizontal reference line represents the upper limit of normal ferritin. Despite persistent marked hyperferritinemia, transferrin saturation remained normal throughout evaluation, arguing against systemic iron overload and supporting a metabolic-inflammatory mechanism of ferritin elevation.

Liver biochemistry revealed mild persistent elevation of alanine aminotransferase, with peak values of 66 U/L. Bilirubin, gamma-glutamyl transferase, and lactate dehydrogenase were within normal limits. C-reactive protein was normal. Renal function was preserved, and there was no evidence of proteinuria.

Metabolic evaluation demonstrated significant insulin resistance with a homeostasis model assessment of insulin resistance (HOMA-IR) of 6.3 and prediabetes with glycosylated hemoglobin (HbA1c) of 6.1%. Triglyceride levels were persistently elevated, reaching up to 4.0 mmol/L. Uric acid levels ranged between 365 and 400 μmol/L.

Serum protein electrophoresis showed a polyclonal pattern with elevated gamma globulins. Total IgG was elevated at 21.6 g/L, with increased IgG1 and IgG2 subclasses.

Autoimmune workup, including extractable nuclear antigen panel, anti-double-stranded DNA antibodies, complement levels, and myositis panel, was negative. Paraneoplastic antibody screening was also negative.

Complete blood counts remained stable with normal hemoglobin and platelet counts. Peripheral smear examination was unremarkable, and hemoglobin electrophoresis was normal. Key serial laboratory findings, metabolic parameters, and immunologic evaluation are summarized in Table 1.

**Table 1:** Serial laboratory, metabolic, and immunologic parameters. Serial laboratory investigations demonstrating metabolic dysfunction, preserved hematologic parameters, and polyclonal hypergammaglobulinemia (“-” indicates parameter not assessed at that time point). ALT: alanine aminotransferase; AST: aspartate aminotransferase; HbA1c: glycosylated hemoglobin; HOMA-IR: homeostasis model assessment of insulin resistance.

Parameter	Sep-25	Dec-25	Apr-26	Reference range
Transferrin saturation (%)	-	-	23	15-50
Total IgG (g/L)	-	-	21.6	7.0-16.0
IgG1 (g/L)	-	-	11.9	3.82-9.29
IgG2 (g/L)	-	-	8.97	2.42-7.00
ALT (U/L)	35	36	66	<35
AST (U/L)	23	23	32	<35
Triglycerides (mmol/L)	2.3	3.9	4	<1.7
HbA1c (%)	5.5	5.9	6.1	<5.7
HOMA-IR	-	-	6.3	<2.0
Uric acid (µmol/L)	388	365	396	140-360
CRP (mg/L)	1.2	1.5	-	0-5
Hemoglobin (g/dL)	14.8	13.9	14.5	12-16
Platelets (x10^9/L)	350	354	381	150-450

Abdominal ultrasonography demonstrated fatty liver without focal lesions. Transient elastography revealed severe steatosis (S3) with liver stiffness of 5.63 kPa, indicating the absence of significant fibrosis. Representative transient elastography imaging is shown in Figure 2.

**Figure 2:**
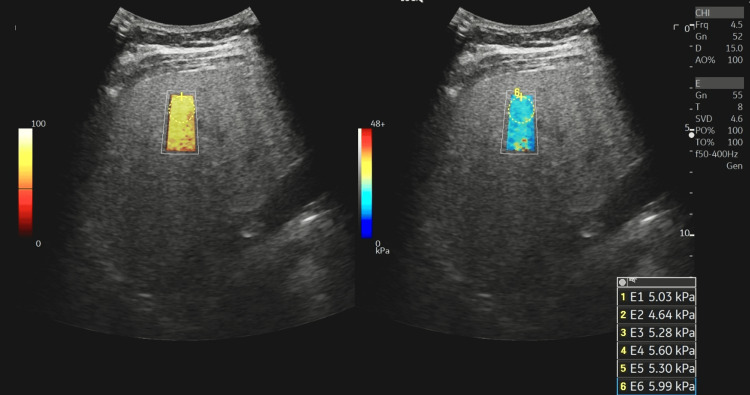
Transient elastography demonstrating severe hepatic steatosis with low liver stiffness. The figure demonstrates transient elastography findings consistent with severe hepatic steatosis (S3) with preserved liver stiffness measurements ranging between 4.64 and 5.99 kPa (median 5.63 kPa), indicating the absence of clinically significant hepatic fibrosis despite marked metabolic dysfunction and persistent hyperferritinemia.

To further evaluate the possibility of iron overload, MRI-based hepatic iron quantification was performed. The liver appeared normal in size with no focal lesions. There was no signal loss on T2-weighted sequences when compared with the spleen and paraspinal muscle. Quantitative analysis demonstrated a liver iron concentration (LIC) of 2.70 mg/g, which was within the normal range. These findings confirmed the absence of hepatic iron overload (Figure 3).

**Figure 3:**
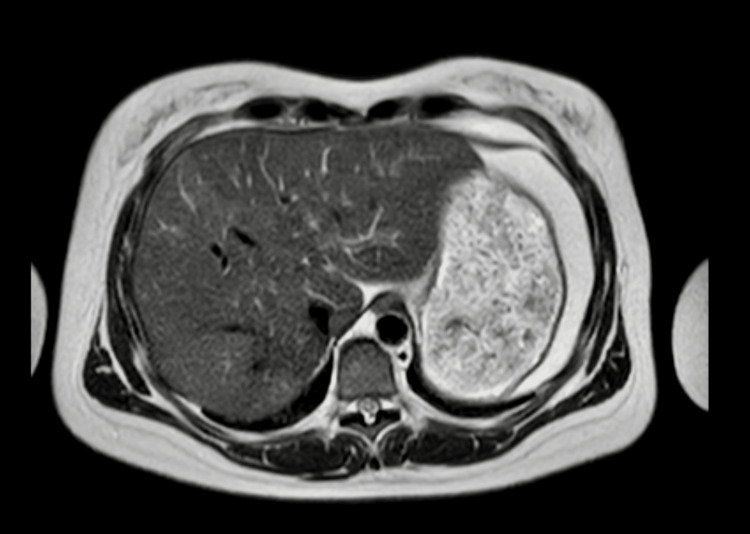
MRI of the liver demonstrating absence of hepatic iron overload. Axial MRI of the liver demonstrating preserved hepatic signal intensity without hypointensity relative to the spleen, consistent with the absence of iron deposition. Quantitative MRI confirmed normal liver iron concentration (LIC = 2.70 mg/g).

## Discussion

Framing the case: A pattern recognition problem, not a rare disease

This case does not represent a rare or novel disease entity; rather, it illustrates a clinically important pattern within the spectrum of MASLD. The patient demonstrated a constellation of findings, including persistent marked hyperferritinemia, normal transferrin saturation, severe hepatic steatosis without fibrosis, insulin resistance with prediabetes, hypertriglyceridemia, hyperuricemia, and polyclonal hypergammaglobulinemia. Each of these abnormalities is individually well described in metabolic disease; however, their coexistence creates a diagnostic scenario that frequently leads clinicians to consider iron overload syndromes, autoimmune conditions, or occult malignancy.

The central significance of this case lies in demonstrating that this entire biochemical and clinical profile can be coherently explained within a unified metabolic-inflammatory framework. Recognizing this pattern allows clinicians to shift from fragmented diagnostic thinking toward a systems-based interpretation, thereby avoiding unnecessary investigations and inappropriate interventions.

Ferritin in MASLD: Moving beyond “Iron Marker” interpretation

Ferritin is traditionally interpreted as a surrogate marker for total body iron stores; however, its biological role extends far beyond iron storage. Ferritin is an acute-phase reactant whose expression is tightly regulated by inflammatory signaling pathways and cellular stress responses. At the molecular level, interleukin-6 (IL-6) activates the JAK-STAT3 signaling pathway, leading to transcriptional upregulation of ferritin heavy chain genes, while tumor necrosis factor-alpha (TNF-α) activates NF-κB signaling, further enhancing ferritin synthesis [4,5].

In MASLD, hepatocellular lipid accumulation results in lipotoxicity, mitochondrial dysfunction, and increased production of reactive oxygen species (ROS). This oxidative stress environment stabilizes ferritin mRNA and enhances its translation through modulation of iron-responsive element/iron regulatory protein (IRE/IRP) interactions, allowing ferritin to function as a cytoprotective molecule that sequesters redox-active iron [6]. Consequently, ferritin elevation in MASLD reflects an integrated response to inflammation and oxidative stress rather than a direct measure of iron excess.

In contrast to hereditary hemochromatosis, where transferrin saturation is typically elevated due to increased circulating iron availability, inflammatory hyperferritinemia in MASLD reflects cytokine-driven ferritin upregulation and intracellular iron sequestration. Similarly, unlike extreme ferritin elevations seen in adult-onset Still disease, hemophagocytic lymphohistiocytosis, or severe systemic inflammatory syndromes, this patient demonstrated preserved hematologic parameters, absence of systemic inflammatory collapse, and stable long-term biochemical findings, supporting a chronic metabolic-inflammatory process rather than acute hyperinflammatory disease.

In this patient, the persistence of ferritin levels between 1281 and 1642 ng/mL over 19 months underscores sustained activation of these pathways, rather than progressive iron accumulation.

Hepcidin-ferritin paradox: Iron sequestration without iron overload

A defining feature of this case is the dissociation between markedly elevated ferritin and normal transferrin saturation (23%). This reflects a state of iron sequestration rather than iron overload and can be explained by dysregulation of the hepcidin-ferroportin axis.

Hepcidin, a peptide hormone produced by hepatocytes, is the principal regulator of systemic iron homeostasis. It binds to ferroportin, the only known cellular iron exporter, inducing its internalization and degradation, thereby reducing iron efflux from hepatocytes and macrophages [7]. In inflammatory states such as MASLD, IL-6-mediated signaling upregulates hepcidin expression, resulting in reduced ferroportin activity and intracellular retention of iron [5].

This leads to increased ferritin synthesis as a storage and detoxification mechanism, while circulating iron levels remain stable, resulting in normal transferrin saturation. This paradox distinguishes MASLD-associated hyperferritinemia from hereditary hemochromatosis, in which hepcidin suppression leads to increased intestinal iron absorption and elevated transferrin saturation [8].

The recognition of this mechanism is critical, as it reframes hyperferritinemia in MASLD as a marker of iron redistribution and inflammatory signaling rather than systemic iron overload.

Integrated immunometabolic activation axis

The phenotype observed in this patient can be conceptualized as an integrated immunometabolic activation axis, in which metabolic dysfunction, hepatic injury, and immune activation are interlinked.

Insulin resistance, as evidenced by a HOMA-IR of 6.3 and HbA1c of 6.1%, promotes increased lipolysis in adipose tissue, resulting in elevated free fatty acid flux to the liver. This leads to hepatic triglyceride accumulation and steatosis, which in turn induces lipotoxic injury and mitochondrial dysfunction. The resultant oxidative stress activates intracellular signaling pathways, including NF-κB and JNK (c-Jun N-terminal Kinase), driving the production of pro-inflammatory cytokines such as IL-6 and TNF-α [9,10].

These cytokines stimulate ferritin synthesis and modulate iron regulatory pathways, while simultaneously promoting systemic inflammatory signaling. In parallel, activation of Kupffer cells and hepatic innate immune pathways leads to downstream activation of adaptive immunity, resulting in polyclonal B-cell activation and increased immunoglobulin production.

Thus, the coexistence of hyperferritinemia and polyclonal hypergammaglobulinemia reflects parallel outputs of a shared immunometabolic network, rather than independent pathological processes. This integrated framework reinforces the concept of MASLD as a systemic disorder extending beyond hepatic fat accumulation.

MRI correlation: Resolving the iron overload diagnostic conflict

A critical component in this case was the integration of MRI-based hepatic iron quantification, which provided objective confirmation of the biochemical interpretation. Despite persistently elevated ferritin levels exceeding 1500 ng/mL, MRI demonstrated normal hepatic signal characteristics without evidence of iron deposition. Quantitative assessment revealed a liver iron concentration of 2.70 mg/g, which lies well within the normal range.

MRI has emerged as a reliable non-invasive modality for assessing hepatic iron content, with T2* and R2* techniques allowing accurate quantification of iron deposition. In iron overload states, paramagnetic effects of iron result in signal loss on T2-weighted sequences and reduced T2 values. The absence of such changes in this patient directly contradicts the hypothesis of iron overload and supports a model of iron sequestration rather than accumulation [11,12].

This radiologic confirmation is particularly important in cases of marked hyperferritinemia, where reliance on ferritin alone may lead to diagnostic misclassification. The concordance between normal transferrin saturation and normal MRI-derived liver iron concentration provides strong evidence against disorders of increased iron absorption or deposition.

Thus, MRI serves not merely as an adjunct investigation but as a decisive tool in resolving diagnostic ambiguity, reinforcing the interpretation of hyperferritinemia as a consequence of metabolic inflammation rather than iron overload.

Diagnostic implications: Structured approach to hyperferritinemia

This case highlights the importance of a structured and physiology-based approach to hyperferritinemia. In patients presenting with ferritin levels exceeding 1000 ng/mL, initial evaluation should prioritize measurement of transferrin saturation to differentiate between iron overload and non-iron overload states.

A normal transferrin saturation, as demonstrated in this patient, argues strongly against disorders characterized by increased intestinal iron absorption, such as hereditary hemochromatosis. In such cases, diagnostic focus should shift toward metabolic, inflammatory, hepatic, or malignant causes.

The presence of metabolic risk factors, including insulin resistance, hypertriglyceridemia, hyperuricemia, and hepatic steatosis, in conjunction with preserved hematologic parameters and normal inflammatory markers, strongly supports a diagnosis of MASLD-associated hyperferritinemia. Recognition of this pattern can prevent unnecessary invasive investigations, such as liver biopsy, and avoid inappropriate therapeutic interventions, including phlebotomy.

Differential diagnosis and exclusion of mimics

Marked hyperferritinemia necessitates careful consideration of several differential diagnoses. Hereditary hemochromatosis is unlikely in the presence of normal transferrin saturation. Ferroportin disease, although capable of presenting with elevated ferritin and normal transferrin saturation, typically demonstrates evidence of iron accumulation and lacks the metabolic context observed in this case.

Autoimmune hepatitis was excluded based on negative serologic testing and absence of characteristic biochemical patterns. Hematologic malignancy and plasma cell disorders were ruled out by the presence of a polyclonal electrophoretic pattern, stable blood counts, and normal peripheral smear. Hyperinflammatory syndromes such as hemophagocytic lymphohistiocytosis or adult-onset Still’s disease were unlikely, given normal C-reactive protein levels, absence of cytopenias, and stable clinical course.

These findings collectively support a metabolic etiology for the observed biochemical abnormalities.

Polyclonal hypergammaglobulinemia as a marker of metabolic immune activation

The presence of polyclonal hypergammaglobulinemia in this patient reflects chronic immune activation rather than antigen-driven autoimmunity. IL-6 plays a central role in promoting plasma cell differentiation and immunoglobulin production, providing a mechanistic link between hepatic inflammation and systemic immunoglobulin elevation.

Unlike autoimmune hepatitis, where immunoglobulin elevation is associated with specific autoantibodies and immune-mediated hepatocellular injury, the immunoglobulin increase observed here represents nonspecific, cytokine-driven B-cell activation. This supports the interpretation of polyclonal hypergammaglobulinemia as a biomarker of metabolic inflammation.

Therapeutic implications and emerging strategies

Metabolic-directed therapy, including metformin, dapagliflozin, fenofibrate, omega-3 fatty acids, and lifestyle intervention, was initiated during the later phase of evaluation following exclusion of primary iron overload and autoimmune liver disease. Consequently, a substantial portion of the biochemical observation period preceded full therapeutic optimization, which may partially explain the persistence of metabolic and inflammatory abnormalities during serial assessment.

The management of this phenotype should focus on addressing the underlying metabolic dysfunction rather than targeting ferritin levels directly. In this patient, treatment with metformin, sodium-glucose cotransporter-2 (SGLT2) inhibition, fenofibrate, and omega-3 fatty acids reflects an appropriate metabolic approach; however, persistent biochemical abnormalities suggest ongoing disease activity.

GLP-1 receptor agonists represent a rational therapeutic escalation, as they improve insulin sensitivity, reduce hepatic steatosis, and modulate inflammatory signaling pathways through activation of AMP-activated protein kinase and related pathways [13]. Emerging therapies, including dual incretin agonists and fibroblast growth factor 21 analogs, offer additional potential by targeting both metabolic and inflammatory pathways [14].

Genetic modifiers and phenotypic variability

Genetic polymorphisms contribute to variability in MASLD phenotype. Variants in PNPLA3 and TM6SF2 have been associated with increased hepatic fat accumulation, inflammation, and fibrosis risk [15]. These variants may influence inflammatory signaling pathways and ferritin expression, potentially contributing to exaggerated biochemical phenotypes, such as that observed in this case, in broader patient populations. However, these variants were not specifically evaluated in the present case.

Learning points

This case highlights that marked hyperferritinemia in MASLD may reflect chronic inflammatory signaling, oxidative stress, and iron sequestration rather than true systemic iron overload. In such patients, transferrin saturation serves as the most important discriminator in differentiating metabolic hyperferritinemia from primary iron overload syndromes such as hereditary hemochromatosis. The coexistence of polyclonal hypergammaglobulinemia further supports the concept of MASLD as a systemic immunometabolic disorder involving persistent cytokine-driven immune activation rather than isolated hepatic fat accumulation alone. Recognition of this biochemical and clinical pattern is essential to prevent misdiagnosis, avoid unnecessary invasive investigations or inappropriate phlebotomy, and guide clinicians toward a metabolism-focused therapeutic strategy.

## Conclusions

This case illustrates a clinically important pattern within MASLD characterized by marked hyperferritinemia and polyclonal hypergammaglobulinemia in the absence of iron overload. These findings reflect an integrated immunometabolic process rather than isolated pathology. The addition of MRI-based iron quantification provides definitive confirmation of this interpretation, allowing clinicians to confidently exclude iron overload. Recognition of this pattern enables accurate diagnosis, avoids unnecessary investigations, and directs management toward metabolic correction.

Clinicians should recognize that marked hyperferritinemia in MASLD does not necessarily indicate true iron overload, particularly when transferrin saturation remains normal. In such patients, a mechanistic interpretation integrating metabolic dysfunction, inflammatory signaling, and quantitative iron assessment may prevent unnecessary invasive investigations and inappropriate phlebotomy-based interventions.
